# Discovering leaf and stripe rust resistance in soft red winter wheat through genome‐wide association studies

**DOI:** 10.1002/tpg2.70055

**Published:** 2025-06-11

**Authors:** John W. Bagwell, Mohamed Mergoum, Madhav Subedi, Suraj Sapkota, Bikash Ghimire, Benjamin Lopez, James W. Buck, Bochra A. Bahri

**Affiliations:** ^1^ Institute of Plant Breeding, Genetics and Genomics University of Georgia, Griffin Campus Griffin Georgia USA; ^2^ Department of Crop and Soil Sciences University of Georgia, Griffin Campus Griffin Georgia USA; ^3^ Cornell Institute of Biotechnology Cornell University Ithaca New York USA; ^4^ USDA‐ARS Small Grains and Potato Germplasm Research Unit Aberdeen Idaho USA; ^5^ Department of Plant Pathology University of Georgia, Griffin Campus Griffin Georgia USA

## Abstract

Leaf rust (LR) and stripe rust (YR), which are caused by *Puccinia triticina* and *Puccinia striiformis*, respectively, are among the most devastating wheat rusts worldwide. These diseases can be managed by using genetically resistant cultivars, an economical and environmentally safer alternative to fungicides. Over 100 and 80 *Lr* and *Yr* resistance genes have been discovered, respectively; however, rust pathogens are overcoming introduced resistance genes in the southeastern United States. Genome‐wide association study has emerged as a valuable tool to identify new LR and YR resistance loci. In this study, a panel of 263 soft red winter wheat genotypes was evaluated for LR and YR severity in Plains, GA, and Williamson, GA, in a randomized complete block design of two replicates during 2019 and 2021–2023. Also, LR and YR infection types were assessed on seedlings at the three leaf stage in three greenhouse trials. A total of 26 significant quantitative trait loci (QTL) explaining 0.6%–30.8% phenotypic variance (PV) was detected by at least two of the five GAPIT models (BLINK, CMLM, FarmCPU, GLM, and MLM) tested. Nine major QTL included *QLrYr‐2A.1* linked to single‐nucleotide polymorphism S2A_20855466, which had the highest overall PV (30.8%) for response to both rust pathogens in the field. Using the Chinese Spring Reference Genome Version 1.0, we detected 16 candidate genes, and four known *R* genes and QTL overlapped two major QTL. Of these QTL, 16 are likely novel genetic loci with potential for marker‐assisted selection.

## Core Ideas

- Genome‐wide association study discovered 26 quantitative trait loci (QTLs) for leaf and stripe rust resistance in soft red winter wheat.
- Of the nine major QTLs, two overlapped four known *R* genes or QTL, like *YrR61*, a *Yr* gene found in the US Southeast.
- Major and minor QTLs overlapped 22 known *R* genes or QTLs in total.

**Abbreviations**
BLUE: best linear unbiased estimates
DS: disease severity
GWAS: genome‐wide association study
IT: infection type
LR: leaf rust
*Pst*
: *Puccinia striiformis*
*Pt*
: *Puccinia triticina*
PV: phenotypic variance
QTL: quantitative trait locus
SNP: single‐nucleotide polymorphism
SWAMP: soft wheat association mapping panel
YR: stripe rust
*Yr* gene: stripe rust resistance gene

## 1 INTRODUCTION

Wheat (*Triticum aestivum* L.) is grown on 217 million ha yearly, and it accounts for 18% and 19% of all dietary calories and protein intake globally, respectively (Erenstein et al., 2022). Soft red winter wheat is the largest class grown in the southeastern United States (Vocke & Ali, 2013). In the 2023/2024 marketing year, soft red winter wheat contributed almost 25% of the total US wheat production (https://www.ers.usda.gov/data‐products/wheat‐data/). Wheat production is seriously impacted by several diseases, including rusts, which can cause up to $5 billion in losses annually worldwide (Figuero et al., 2018). Leaf (brown) rust (LR), caused by *Puccinia triticina* Eriks (*Pt*), is the most widespread of the wheat rusts (Bolton et al., 2008), and it has been projected to cause up to $3.3 billion/year in losses worldwide until 2050 (Chai et al., 2020). Stripe (yellow) rust (YR), caused by *Puccinia striiformis* Westend (*Pst*), can cause total yield loss on susceptible cultivars, especially if infection occurs early and continues to worsen throughout the growing season (Chen, 2005). An estimated 88% of the wheat varieties grown around the world are YR susceptible (Beddow et al., 2015). YR accounted for over 3.17 million metric tons in US wheat losses in 2016, and Pacific Northwest wheat growers may spend at least $10 million annually to manage YR (Chen, 2020). From 2008 to 2019, production of soft red winter wheat in the southeastern United States was reduced by approximately 61%, due in part to LR and YR damages (Ghimire et al., 2020).

The most common options to manage LR and YR include use of synthetic fungicides (Buck et al., 2011; Carmona et al., 2020), cultural controls (Roelfs et al., 1992), and genetic resistance (Sapkota et al., 2020; Ward et al., 2021). Host resistance is the most cost‐effective and ecologically friendly control option. Developing LR‐resistant wheat varieties provides an approximate 27:1 benefit–cost ratio (Kolmer et al., 2009), and high‐temperature adult plant YR resistance has protected wheat in the western United States from millions of dollars in yield losses (Line et al., 1995). Two types of wheat rust resistance include all‐stage resistance (Bariana et al., 2022; Chen, 2005) and adult‐plant resistance (R. P. Singh et al., 2011). Qualitative resistance genes provide race‐specific vertical resistance in a gene‐for‐gene relationship with a rust avirulence gene for all‐stage resistance throughout the life of the plant (L. Liu et al., 2018). However, all‐stage resistance might only be effective for a short duration since the new pathogen races could easily overcome this single‐gene resistance. Adult plant resistance, usually controlled by multiple minor genes providing horizontal non‐race‐specific resistance, starts at adult plant stages and is typically more durable (Riaz et al., 2016). Varieties with adult plant resistance against YR can express that resistance after Feekes stage 6 (Kleczewski et al., 2020; Large, 1954). For YR, one gene expressing high temperature adult plant resistance usually adds little to no *Pst* race selection pressure due to its partial, non‐race‐specific resistance. Also, cultivars should be able to express enough high temperature adult plant resistance to help them overcome a wet, cool environment acting against that resistance gene expression in favor of *Pst* growth (Liu et al., 2018). A combination of all‐stage resistance and adult plant resistance genes provides a more comprehensive resistance to rust diseases (L. Liu et al., 2018; Oelke & Kolmer, 2005).

Over 100 LR resistance (*Lr*) genes and 80 YR resistance (*Yr*) genes have been identified (Mapuranga et al., 2022). However, over 50 new *Pt* races are detected in the United States yearly (Kolmer et al., 2007), and newer *Pst* races, with better urediniospore germination and briefer latency, have acclimated to hotter climate, such as in the southern United States (Milus et al., 2009, 2006; Ward et al., 2021). Most leaf rust resistance (*Lr*) genes are race‐specific and provide all‐stage resistance, so *Pt* populations with significant mutation and diverse virulence can quickly overcome these genes (Pinto da Silva et al., 2018). *Yr17*, an all‐stage resistance gene tightly linked with *Lr37* and *Sr38* (Bariana & McIntosh, 1993), has been used by many wheat breeding programs for rust resistance (Ward et al., 2021). However, 88% of 235 *Pst* isolates from around the world expressed virulence against *Yr17* (Sharma‐Poudyal et al., 2013). PSTv‐37, which has been a predominant US *Pst* race, has overcome *Yr17* (L. Liu et al., 2018). Thus, novel sources of resistance are needed to manage these two rust pathogens.

Doubled haploid (Lu et al., 2017; Prins et al., 2011; Ramburan et al., 2004) and recombinant inbred line populations have been used to identify novel sources of rust resistance (B. Bai et al., 2022; Sapkota et al., 2018, 2020; M. Yang et al., 2019). Diversity panels have also been assembled to conduct genome‐wide association studies (GWAS) for the detection of rust resistance quantitative trait loci (QTLs). Diversity panels are usually used for genetic mapping at higher resolution due to more recombination events compared to biparental populations, and these populations can be assembled more quickly (Baranwal, 2022; Sapkota et al., 2019). Wheat diversity panels have been investigated using DArT, DArT‐Seq (Jighly et al., 2016), 9K (Maccaferri et al., 2015; Turner et al., 2017), and 90K single‐nucleotide polymorphism (SNP) arrays (Gao et al., 2016; Pasam et al., 2017) for high‐throughput genotyping to find new rust‐resistance alleles (Baranwal, 2022). Sapkota et al. (2019) and Ward et al. (2021) have recently found novel sources of resistance to LR and YR from diversity panels, respectively, that can be useful for the southeastern United States. New alleles need to be validated prior to use in breeding programs, including via physical positioning and recombinant inbred line populations. Just over 20 out of the at least 240 known wheat rust resistance genes have been isolated with accessible genomic resources due to issues such as wheat genome complexity (Baranwal, 2022).

A soft wheat association mapping panel (SWAMP) of 263 soft red winter wheat elite lines and released cultivars developed by wheat breeding programs in the southeastern United States (SunGrains; http://www.sungrains.lsu.edu/about.shtml) was evaluated for agronomic traits (Pradhan et al., 2019; Subedi et al., 2024), physiological traits related to heat response (Pradhan et al., 2020), and Fusarium head blight resistance (Ghimire et al., 2022). Ghimire et al. (2022) discovered 11 major QTLs within the SWAMP using GWAS that could collectively reduce Fusarium head blight symptoms by up to 55% by pyramiding resistance alleles. Using the same diversity panel, Pradhan et al. (2020, 2019) were able to find marker trait associations linked with soft wheat spike fertility at high temperatures and stable grain yield under heat stress. The SWAMP panel was used in the current study to decipher the genetics of LR and YR resistance in soft red winter wheat and to find novel resistance loci that could be used by breeding programs. The objectives of this study were to (1) evaluate the panel for LR and YR resistance at seedling stage in the greenhouse and at adult stage under field conditions at the Plains and Williamson experiment stations in Georgia and (2) identify novel QTL for LR and YR resistance using GWAS.

**Core Ideas**

- Genome‐wide association study discovered 26 quantitative trait loci (QTLs) for leaf and stripe rust resistance in soft red winter wheat.
- Of the nine major QTLs, two overlapped four known *R* genes or QTL, like *YrR61*, a *Yr* gene found in the US Southeast.
- Major and minor QTLs overlapped 22 known *R* genes or QTLs in total.

## 2 MATERIALS AND METHODS

### 2.1 Plant materials

The SWAMP panel used in this study included 263 soft red winter wheat elite lines and varieties released (https://www.ncbi.nlm.nih.gov//bioproject/PRJNA578088) by private companies and public wheat breeding programs in the southeastern United States region, particularly SunGrains (University of Arkansas, University of Florida, University of Georgia (UGA), Texas A&M University, North Carolina State University, Louisiana State University, and Clemson University) and Virginia Polytechnic Institute and State University (Pradhan et al., 2020) (Table S1). In 2019, AGS 3030 (LR and YR resistant) (Mergoum et al., 2021b) and SS520 (LR and YR susceptible) (Sapkota et al., 2020; Ward et al., 2021) were included as checks. All other studies used AGS 2024 (LR and YR resistant) (Mergoum, Johnson, Buck, Sutton, Lopez, Bland, Chen, Buntin, Mailhot, Babar, Mason, Harrison, Murphy, Ibrahim, Sutton, Brown‐Guedira, et al., 2021), Hilliard (moderate LR resistance, YR susceptible at seedling stage, YR high temperature adult plant resistance) (Griffey et al., 2020), GW2032 (LR and YR resistant) (Mergoum et al., 2021a), and USG 3555 (LR susceptible) (Martinez et al., 2014) as checks.

### 2.2 Field experimental design

Field trials were conducted at the UGA Southwestern Research and Education Center (SWREC) in Plains, GA (32.05° N, −84.37° W) and Bledsoe Research Farm in Williamson, GA (33.17° N, −84.41° W). The SWREC has Greenville sandy clay loam soil (Mailhot, 2018), and the Bledsoe Research Farm has Cecil sandy loam soil (Gassett, 2015). Two replicates for each entry of the SWAMP panel were laid out in a randomized complete block design. In the 2021 growing season, each entry was planted in two adjacent 1‐m long rows. In growing seasons 2022–2023, each entry was planted in only one 1 m long row. Checks were alternated every 20th row. The experiments were planted in November of each year and irrigated as needed to assure proper germination and crop establishment. Pre‐plant fertilizer was added at a rate of 459.46 kg ha^−1^ annually in October or November. 22.97 kg of urea nitrogen (N) ha^−1^, 20.17 kg of potassium (K) ha^−1^, and 57.15 kg of phosphorus (P) ha^−1^ were applied from this fertilizer rate. Zidua (BASF), a granular pre‐emergence herbicide, was applied at 46.23 g ha^−1^, and ProwlH_2_O, a broad‐spectrum, residual herbicide (BASF), was added at 2.24 kg ha^−1^ in early November. In early February, all plots were top‐dressed with 36.28 kg of liquid urea ammonium nitrate at a rate of 211.37 L ha^−1^, and broadleaf herbicide Harmony Extra (FMC) was applied at a rate of 146.15 mL ha^−1^.

Rust susceptible USG 3555 and SS520 were planted around the experiment. *Pt* race MFGKG is a common race in Georgia (Sapkota et al., 2020), and PSTv‐52 is a *Pst* race prevalent throughout the United States (Q. Bai et al., 2021). SS520 seedlings inoculated, according to protocol found in Section 2.3, with either *Pt* race MFGKG or *Pst* race PSTv‐52 were transplanted in the fields in the middle of the USG 3555 and SS520 spreader plots around the field during early spring, when environmental conditions were most ideal for rust development, to supplement natural field rust epidemics (Bouwman et al., 1985; Fajolu, 2023; Martinez et al., 2009). Seedlings were at least 10 days old with secondary leaves fully opened (Sapkota et al., 2018). Natural LR infection was the only source of LR inoculum in 2021–2022, and there was no YR inoculation in 2019.

### 2.3 Greenhouse experimental design

The SWAMP panel was used for greenhouse experiments at the UGA Griffin campus. Plants were grown in cone‐shaped pots (3.81 × 10.16 cm) in 98 cone‐tainer racks (Stuewe and Sons, Inc.) with Pro‐Mix (Pro‐Mix Gardening) growing mix in a greenhouse at 18°C–24°C with average photoperiod of 11.23, 11.98, 13.02, 13.53, 12.26, and 11.59 h for February–May 2022 and September‐October 2023, respectively (https://www.wunderground.com/). Every 79 L of Pro‐Mix growing mix was amended with 96.9 g of Pennington ProCare Premium All‐Purpose Fertilizer 10‐10‐10 (Pennington Seed, Inc.), 123.9 g of MicroMax Granular G90505 (Custom Hydro), and 116.9 g of Pennington Fast Acting Lime (Pennington Seed, Inc.). Three seeds of a check or entry were planted in each cone. Osmocote Bloom 12‐7‐18 fertilizer (ICL Fertilizers) was added into the potting mix once seedlings had emerged. Each line had three replicates laid out in a randomized complete block design. The greenhouse 2022 LR study had two dates for data collection, both of which were used for data analyses, and the 2023 LR study and 2024 YR study each had one date for data collection. Cones were distributed so that each rack, except for the last rack of each replicate, had 42 entries. All racks were kept in tubs filled with water, and every 2 weeks after applying Osmocote Bloom, or when the plants started to show nutrient deficiency symptoms, plants were fertilized with Jack's Professional Water‐Soluble Fertilizer 20‐20‐20 (JR Peters, Inc.) at 1500 ppm of N 5 L^−1^ of water.

In spring 2022 before the first date of data collection, frozen urediniospores of the LR race MFGKG (Sapkota et al., 2018) were heat shocked between 40°C and 45°C for 10 min (Skoppek & Streubel, 2023) and suspended in 200 mL water + 0.01% Tween‐20 at 10^5^ spores mL^−1^ (Buck, 2013; Sapkota et al., 2018). The spore suspension was applied to 10‐day‐old seedlings (Feekes stage 1.2) (Acevedo et al., 2002) with a spray bottle until runoff, and plants were placed for 48 h in dew chambers in the dark with high humidity. The inoculation was repeated again after 24 h and seedlings were returned to their original greenhouse benches (Sapkota et al., 2018) at 18°C–24°C. In spring 2022 for the second date of data collection and Fall 2023, inoculum for *Pt* race MFGKG was a combination of 10^5^ fresh spores mL^−1^ (vacuumed from SS520 seedlings) and 2 × 10^5^ frozen spores mL^−1^.

For YR, seedlings were inoculated similarly to the LR experiment with fresh spores of PSTv‐52 (Q. Bai et al., 2021). The panel was sprayed with spore suspension until runoff, placed in dew chambers to maximize the humidity, and then transferred to growth chambers (Conviron) for 24 h in complete darkness at around 10°C, and then returned to their original greenhouse benches at around 20°C (Hao et al., 2011; Sapkota et al., 2019). Average photoperiod was 9.92, 10.19, and 10.62 h for December 2023–February 2024, respectively (https://www.wunderground.com/).

### 2.4 Data collection

YR and LR phenotypic data were collected on the field experiment in the 2019, 2021, 2022, and 2023 growing seasons, except for YR in the 2019 growing season and LR in the 2022 growing season. Data collection for disease severity (DS) for each disease was expressed as percentage of flag leaf tissue that was infected and was scored on a 0%–100% modified Cobb scale (Roelfs et al., 1992; Sapkota et al., 2020). DS from adult plant flag leaf was collected in April/May at Feekes stage 10–11 (Large, 1954).

For greenhouse experiments (G22_D1 = first date of data collection for greenhouse 2022 experiment, G22_D2 = second date of data collection for greenhouse 2022 experiment, G23 = greenhouse 2023 experiment), reactions to LR were scored when most susceptible checks expressed significant LR infection symptoms. Infection type (IT) was classified using a Stakman scale (Stakman et al., 1962): 0 = no uredia; = flecking; 1 = small, necrotic uredia; 2 = small to medium uredia with chlorosis or necrosis; 3 = medium uredia with or without chlorotic areas; and 4 = large, non‐chlorotic uredia. Minus and plus signs represented variation in an IT (Roelfs et al., 1992). If a leaf had multiple ITs, one comma separated discrete IT, with the most prevalent IT put first (i.e., 3,1). If a leaf had IT range, values were concatenated together, with the most predominant IT first (i.e., 31) (Roelfs et al., 1992). For values like “;” and more than one IT on one plant, ITs were transformed into a linearized scale of 0–9 (Zhang et al., 2014). Ratings of 0–6 were considered resistant and 7–9 susceptible (Kertho et al., 2015; Sapkota et al., 2019).

YR IT in the greenhouse (G24 = greenhouse 2024 experiment) was rated 3 weeks after inoculation when most susceptible checks expressed good YR infection symptoms. A 0–9 scale was used (McNeal et al., 1971): 0 = no infection seen; 1 = chlorotic or necrotic flecking with no sporulation; 2 = chlorotic or necrotic stripes with no sporulation; 3 = chlorotic or necrotic stripes with trace amounts of sporulation; 4 = chlorotic or necrotic stripes with light sporulation; 5 = chlorotic or necrotic stripes with intermediate sporulation; 6 = chlorotic or necrotic stripes with moderate sporulation; 7 = chlorotic or necrotic stripes with plentiful sporulation; 8 = chlorosis with plentiful sporulation; and 9 = plentiful sporulation with no chlorosis (McNeal et al., 1971). A “–” sign was used if a rated leaf had a range of IT, with the most prevalent IT going first (i.e., 5–3). Discrete ITs on the same leaf were separated by comma, with the most prevalent IT going first (i.e., 5,1) (Milus et al., 2015). ITs of 0–4 were considered resistant and 5–9 were considered susceptible (Wan & Chen, 2012).

### 2.5 Phenotypic data analysis

Phenotypic data analysis for field and greenhouse data was performed in R version 4.3.2 (Posit Software, PBC). A frequency distribution for each trait (DS and IT) was drawn to show potential segregation among lines for disease reactions. Pearson correlations (*r*) were calculated to detect pairwise relationships between traits. Analysis of variance (ANOVA) was utilized for analyzing differences between dependent variables (DS and IT) and their interactions. The Shapiro–Wilk test and Levene's test were utilized to evaluate trait normality with the dplyr R package (Yarberry, 2021) and homogeneity with the car R package, respectively (Fox et al., 2012). Levene's test was utilized because it is less sensitive to non‐normal data. Fisher's least significant difference (LSD) test helped to observe differences between genotypes. All 263 genotypes were used for these analyses as well as heritability (Table S2).

Broad sense heritability (*H* ^2^) was calculated for all traits using the lme4 package in R (Bates et al., 2015). If all data were homogeneous and/or normal, Equation (1) was used (Ghimire et al., 2022; Sapkota et al., 2020; Winn et al., 2021):

(1) $$H^{2}=\frac{\sigma_{G}^{2}}{\sigma_{G}^{2}+\frac{\sigma_{G\;\times \;E}^{2}}{e}+\frac{\sigma_{G\;\times \;Y}^{2}}{y}+\frac{\sigma_{G\;\times \;E\;\times \;Y}^{2}}{e\;\times \;y}+\frac{\sigma_{e}^{2}}{e\;\times \;y\;\times \;r}\;}$$

where *σ* ^2^_G_ is the genotypic variance, *σ* ^2^_G × E_ is the genotypic by environment (G × E) variance, *σ* ^2^_G × Y_ is the genotypic by year (G × Y) variance, *σ* ^2^_G × E × Y_ is the G × E by year (G × E × Y) variance, *σ* ^2^_e_ is the variance from error, *r* is the number of replications, *e* is the number of environments, and *y* is the number of years. For heterogeneous data and not normal datasets, Equation (2) was applied (Ghimire et al., 2022; Winn et al., 2021):

(2) $$H^{2}=\frac{\sigma_{G}^{2}}{\sigma_{G}^{2}+\frac{\sigma_{e}^{2}}{r}\;}$$

The lme4 software package in R (Bates et al., 2015) was also used to calculate best linear unbiased estimates (BLUE) with genotype as a fixed effect and all other variables as random effects. BLUEs were calculated separately and combined for field plots, depending on which datasets were homogeneous or normal when evaluated together (Table S3).

The datasets were named as P19_LR (Plains 2019 LR), P21_LR (Plains 2021 LR), P21_YR (Plains 2021 YR), W21_LR (Williamson 2021 LR), W21_YR (Williamson 2021 YR), P22_YR (Plains 2022 YR), W22_YR (Williamson 2022 YR), P23_LR (Plains 2023 LR), P23_YR (Plains 2023 YR), W23_LR (Williamson 2023 LR), W23_YR (Williamson 2023 YR), G22_D1 (Greenhouse 2022 date one LR), G22_D2 (Greenhouse 2022 date two LR), G23 (Greenhouse 2023 LR), and G24 (Greenhouse 2024 YR).

### 2.6 Genotyping and population structure

Genotyping and population structure of the panel were performed by Pradhan et al. (2019). Briefly, genotyping by sequencing libraries were created from PstI‐HF and MspI restriction enzymes, pooled in 96‐plex, and sequenced utilizing an Ion Torrent Proton sequencer. TASSEL was used to call SNPs, the International Wheat Genome Sequencing Consortium genome assembly was used to align reads, and markers were filtered so that only markers with minor allele frequency > 5% and < 20% missing data were used for genetic analysis (Pradhan et al., 2019). Analyses for population structure and principal components (PCs) were performed using R packages adegenet and stats by Pradhan et al. (2019). Pradhan et al. (2019) has previously discussed the distribution of markers across the SWAMP panel chromosomes and genomes as well as the PC analysis. Three distinct genetic groups were discovered in the SWAMP panel (Pradhan et al., 2019).

### 2.7 GWAS and linkage disequilibrium

R package GAPIT version 3.0 (v3.0) models General Linear Model (GLM), Mixed Linear Model (MLM), Compressed MLM (CMLM), Fixed and random model Circulating Probability Unification (FarmCPU), and Bayesian‐information and linkage disequilibrium (LD) Iteratively Nested Keyway (BLINK) were used to conduct GWAS (Lipka et al., 2012). GWAS was conducted with 16 BLUE datasets to confirm significant SWAMP marker trait associations in R version 4.3.2. The first three PCs served as covariates according to quantile–quantile and scree plot results. These plots were to check model fit, and PCs can help control for population structure. A kinship matrix was produced in FarmCPU (Pradhan et al., 2019). SNPs were considered significant if their −log_10_(*p*)‐values were higher than the value given from a Bonferroni correction threshold:

(3) $$-\mathrm{lo}g_{10}\frac{p-\mathrm{value}}{\mathrm{number}\;\mathrm{of}\;\mathrm{SNPs}\;\mathrm{in}\;\mathrm{genetic}\;\mathrm{data}}=6.44$$

where *p*‐value = 0.01, and the number of SNPs in the genetic data is 27,466. This threshold was chosen to control the false positive hits since almost all chromosomes had a SNP that was considered significant under a lower threshold of −log_10_(*p*) = 4 (Pradhan et al., 2019). The 10% phenotypic variance (PV) was our threshold for major QTL (Ghimire et al., 2022; Subedi et al., 2024). A plot of the wheat chromosomes was produced using the karyoploteR R package (Gel & Serra, 2017).

In this study, TASSEL v5.0 was used to obtain pairwise LD estimations of SNPs as squared allele frequencies (*r* ^2^) (Bradbury et al., 2007; Hill & Weir, 1988). A sliding window size of 50 was used as an LD parameter (Otyama et al., 2019). LD values were used to visualize rate of LD decay against physical base pair distance in base R (Hill & Weir, 1988). The *r* ^2^ critical value was considered half total LD decay, the intersection of *r* ^2^ and the curve of locally weighted polynomial regression (LOESS) (Roncallo et al., 2021). LD was used to calculate individual *r* ^2^ critical values (Table S4) for each chromosome, genome, and the whole genome for comparison. SNPs were considered linked and belonged to the same QTL block if their pairwise estimates surpassed *r* ^2^ from the whole genome (*r* ^2^ = 0.33) (Otyama et al., 2019).

### 2.8 Allele pyramiding and candidate gene search

Allelic effect was analyzed from major QTL to determine which alleles were associated with lower DS or IT or higher rust resistance. Tukey's honest significant difference test was utilized to evaluate allelic effect of pyramided QTL (Ghimire et al., 2022; Subedi et al., 2024). The qqman R package was used to make Manhattan plots (Turner, 2014). The Chinese Spring reference genome v1.0 was used to find candidate genes associated with significant marker trait associations and their annotation (Appels et al., 2018). Previous literature was used to investigate genes potentially associated with detected QTL (Pradhan et al., 2019; Subedi et al., 2024). A 5 kb range was used to search for candidate genes on Ensembl Plants (Subedi et al., 2024). Sequences of known *Lr* and *Yr* genes and QTL on chromosomes with statistically significant SNPs, or cosegregating marker primers, were run through the Chinese Spring v1.0 basic local alignment search tool (IWGSC BLAST [inrae.fr]) to compare their physical bp positions to detected QTL considered statistically significant and confirm if they are novel.

## 3 RESULTS

### 3.1 Phenotypic data analysis

Field and greenhouse data for all traits deviated from a normal distribution (Figures S1–S3). Maximum LR DS was 93.3% from P23_LR. Plains 2019 had the highest DS field average for LR (31.6%) (Table 1). For LR DS LSD significance groups, 10 lines were in the lowest significance groups in Plains across years, while 28 lines were in the lowest significance group in Williamson across years (Table S5). Correlations across years in Plains for LR DS data ranged from 0.31 to 0.43, and correlation across years for LR DS in Williamson was 0.09 (Table S6). Across all LR DS data, seven out of 10 correlations were statistically significant (*p* < 0.05), and ANOVA showed “genotype” was significant for all but W21_LR (Table S7). Field LR *H* ^2^ ranged from 0.48 to 0.90, and P19_LR had the highest *H* ^2^ (0.90) (Table 1).

**TABLE 1:** Summary statistics and heritability values for leaf rust and stripe rust disease severity and infection type for the soft wheat association mapping panel (SWAMP) diversity panel across environments.

Disease	Year	Location in GA	TEY	Max	Min	SE	Avg	*H* ^2^
**Leaf rust**	**2019**	**Plains**	P19_LR	90.00	5.00	1.47	31.60	0.90
	**2021**		P21_LR	90.00	0.00	0.83	11.35	0.48
		**Williamson**	W21_LR	80.00	0.00	0.22	1.07	0.00
	**2022 D1**	**Greenhouse**	G22_D1	8.00	0.00	0.08	1.37	0.62
	**2022 D2**		G22_D2	8.00	0.00	0.15	2.67	0.86
	**2023**	**Plains**	P23_LR	93.33	0.00	0.69	5.99	0.85
		**Williamson**	W23_LR	40.00	0.00	0.17	1.57	0.51
		**Greenhouse**	G23	8.44	0.00	0.12	2.90	0.78
	**Combined greenhouse environment**		GCE	8.44	0.00	0.05	2.32	0.85
**Stripe rust**	**2021**	**Plains**	P21_YR	80.00	0.00	0.81	14.02	0.53
		**Williamson**	W21_YR	70.00	0.00	0.23	5.59	0.00
	**2022**	**Plains**	P22_YR	90.00	5.00	1.05	31.92	0.72
		**Williamson**	W22_YR	90.00	5.00	1.04	39.17	0.77
	**2023**	**Plains**	P23_YR	70.00	0.00	0.75	9.05	0.80
		**Williamson**	W23_YR	76.67	0.00	0.60	4.65	0.71
	**2024**	**Greenhouse**	G24	8.00	1.00	0.06	3.68	0.36

*Note*: TEY: Trait environment year combination; Max: maximum disease severity value (on a 0%–100% modified Cobb scale [Roelfs et al., 1992; Sapkota et al., 2020]), LR infection type (linearized on a 0–9 scale [D. Zhang et al., 2014]), or YR infection type (on a 0–9 scale [McNeal et al., 1971]); Min: minimum recorded value of disease severity or infection type; SE: standard error; Avg: Average; *H* ^2^: broad sense heritability calculated with Equations (1) and (2) (Ghimire et al., 2022; Sapkota et al., 2020; Winn et al., 2021). 2022 D1 and 2022 D2 for greenhouse 2022 data correspond to the first and second times when data were collected on the soft wheat association mapping panel seedlings in the greenhouse for LR IT in April 2022 and May 2022, respectively. All field datasets had two replicates each, and all greenhouse datasets had three replicates each. GCE: Greenhouse combined environment.

Maximum YR DS was 90% from both P22_YR and W22_YR. Williamson had the highest YR DS average (39.2%) (Table 1). For LSD significance groups for YR DS, ARLA07084C‐10‐1 was the only variety to be found in the lowest significance group for five datasets, including P21_YR, W21_YR, P22_YR, P23_YR, and W23_YR (Table S5). Correlations across years in Plains for YR DS data ranged from 0.20 to 0.44, and correlations across years for YR DS in Williamson ranged from 0.02 to 0.19. The highest correlation for YR DS data were between P22_YR and W22_YR (0.60). Of the 15 correlations for YR DS datasets, nine were statistically significant (*p* < 0.05) (Table S6). ANOVA showed that, just like for LR DS data, “genotype” was significant for all YR DS datasets except W21_YR (Table S7). Field YR *H* ^2^ ranged 0.36–0.80, with P23_YR having the highest *H* ^2^ (0.80) (Table 1).

The highest LR and YR IT values from seedlings were 8.44 and 8, respectively (Table 1). For LSD significance groups, nine and one lines were in the lowest significance group across LR IT datasets and for YR IT data, respectively. The one line in the lowest significance group for YR IT data were 11656‐17E11 (Table S5). Correlations across LR IT datasets ranged from 0.66 to 0.78. The highest correlation between LR and YR IT data were between G22_D2 and G24 (0.37). All correlations across IT datasets were statistically significant (*p* < 0.05) (Table S6). Greenhouse *H* ^2^ was up to 0.86 and 0.36 for LR and YR, respectively (Table 1).

### 3.2 Linkage disequilibrium

Half LD decay distance for the whole genome was 339 Kb, and the D genome had the shortest LD decay distance (∼98 Kb), followed by the A and B genomes (173 and 567 Kb, respectively). Chromosome 2B had the longest decline (6.20 Mb), while chromosome 6D had the shortest decline (55 bp) (Table S4).

### 3.3 Genome‐wide association studies

**FIGURE 1:**
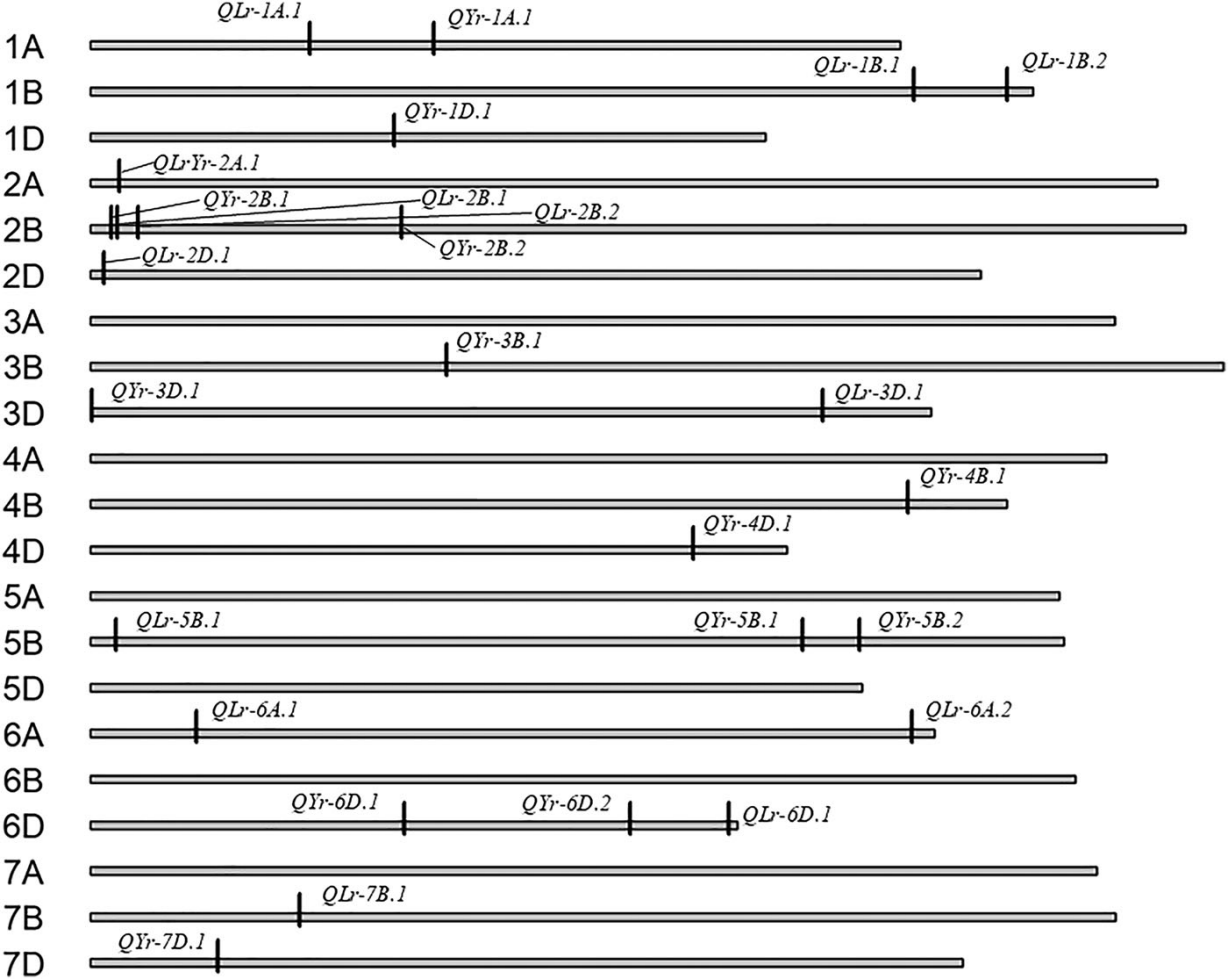
Plot of the wheat chromosomes with labels showing the dataset for which each quantitative trait locus (QTL) was detected. Black tick marks on the chromosomes indicate the location of the QTL. Then *x*‐axis represents the length of each chromosome, and the *y*‐axis represents each chromosome.

A total of 26 significant SNPs (−log_10_(*p*) > 6.44) underlying 26 QTLs were discovered from at least two GWAS models across all datasets (Figure 1). Each QTL only had one significant SNP (Table S8). Out of the 26 QTLs identified, nine were major QTLs with PV > 10% and were detected on chromosomes 1A (*QYr‐1A.1*), 1B (*QLr‐1B.1*), 2A (*QLrYr‐2A.1*), 2B (*QLr‐2B.1*, *QLr‐2B.2*), 2D (*QLr‐2D.1*), 3D (*QYr‐3D.1*), 6A (*QLr‐6A.2*), and 7B (*QLr‐7B.1*). *QLrYr‐2A.1* was pleiotropic and detected from P19_LR, P22_YR, and W22_YR (Table 2). Two major QTLs, *QLrYr‐2A.1* and *QLr‐2D.1*, were found in multiple datasets (Table 2). Four, three, and three major QTLs were detected from P_LR, greenhouse LR, and P_YR data, respectively (Table 2). The highest number of QTLs was detected from LR (7) and YR (6) at P23. Chromosome 2B harbored the most QTLs (4), explaining up to 15.7% of PV, followed by chromosomes 5B and 6D, with three QTLs each, explaining up to 9.8% and 5.95% PV, respectively. The highest PV was detected from *QLrYr‐2A.1* (30.8%), followed by *QLr‐1B.1* (23.8%), *QLr‐2D.1* (18.6%), and *QYr‐3D.1* (16.5%). The highest and lowest allelic effects for LR came from *QLr‐6A.2* (6.3) and *QLrYr‐2A.1* (−15.3), respectively. The highest and lowest allelic effects for YR came from *QYr‐1D.1* (6.98) and *QLrYr‐2A.1* (−9.8), respectively. Genome B had the most QTL (12), followed by genome D (9) and genome A (5). Overall, detected SNPs were considered statistically significant in a dataset 30 times. Across those 30 instances, CMLM detected a significant SNP 17 times; BLINK and MLM both detected a significant SNP 20 times; and FarmCPU and GLM both detected a significant SNP 23 times. For major QTL, FarmCPU and GLM each detected significant SNPs 12 times, which was more than the other three models. Since BLINK, CMLM, and MLM detected significant SNPs fewer times, they may be more conservative models for our data (Table S8).

**TABLE 2:** Nine major quantitative trait loci (QTLs) with phenotypic variance over 10% for leaf rust and stripe rust response detected in the soft wheat association mapping panel (SWAMP).

QTL	SNP^a^	TEY	Allele	MAF	Allelic effect	PV (%)	Gene‐ID	Annotation	Function	Known *R* genes/QTL	Reference
*QYr‐1A.1*	S1A_251376894	P23_YR	**C**/T (204:11)	0.08	4.85	14.99	*TraesCS1A02G145300*	GDSL‐like lipase/esterase	Lipid metabolic process, hydrolase activity	*YrHa*	(Dash et al., 2016; Ma et al., 2013; X. Yang et al., 2022)
*QLr‐1B.1*	S1B_602752224	G23	G/T (189:0)	0.17	−0.89 to −0.86	23.84					
*QLrYr‐2A.1*	S2A_20855466	P19_LR	C/**T** (119:78)	0.41	−15.3 to −13.71	30.76	*TraesCS2A02G052500*	Lysine histidine transporter, enzyme	sulfur deficiency response, effector, nematode response	*YrR61*, *Lr17a*, *Qyrtm.pau‐2A*	(Bremenkamp‐Barrett et al., 2008; Chhuneja et al., 2008; Hao et al., 2011; Marella et al., 2013; Wipf et al., 2014; Yoo et al., 2020)
		P22_YR	C/**T** (119:78)	0.41	−9.83 to −8.12	27.45					
		W22_YR	C/**T** (119:78)	0.41	−9.25 to −5.44	15.86					
*QLr‐2B.1*	S2B_19449473	P23_LR	**G**/C (177:8)	0.13	−4.46	15.74	*TraesCS2B02G042700*	Disease resistance protein group; NB‐ARC domain‐containing protein; CC‐NBS‐LRR *R* protein	Related to potato late blight resistance protein R1; immune response		(Ballvora et al., 2002; Kim et al., 2011)
*QLr‐2B.2*	S2B_34798540	G22_D1	G/**A** (119:19)	0.28	0.74 to 0.80	11.67					
*QLr‐2D.1*	S2D_9629191	G22_D2	C/**T** (134:2)	0.21	−2.53 to −1.54	18.58	*TraesCS2D02G020100*	Apoplastic protein; antifungal protein	Salt stress response in roots in rice; slow growth of fungal pathogens		(Sawano et al., 2007; L. Zhang et al., 2009)
		G23	C/**T** (134:2)	0.21	−1.01	6.86					
		GCE	C/**T** (134:2)	0.21	−1.59 to −1.40	14.46					
*QYr‐3D.1*	S3D_973973	P23_YR	**G**/C (192:15)	0.12	−3.98 to −4.61	16.52					
*QLr‐6A.2*	S6A_601222639	P23_LR	**A**/G (194:18)	0.12	4.89 to 6.34	13.26	*TraesCS6A02G381800*	W2 domain‐containing protein	Decreased expression during temperature, salt, and drought stress		(Habibpourmehraban et al., 2022)
*QLr‐7B.1*	S7B_152782085	P23_LR	**A**/C (147:44)	0.28	2.93 to 3.14	10.09	*TraesCS7B02G127800*	Myb‐like DNA‐binding domain family	Biotic stress response, Lower expression for stomata control under heat stress		(Lau et al., 2018; Sood et al., 2014)

*Note*: SNP: single‐nucleotide polymorphism; TEY: trait‐environment‐year combination (P = Plains, G = greenhouse, W = Williamson, 19 = 2019, 22 = 2022, 23 = 2023, YR = stripe rust, LR = leaf rust), MAF: minor allele frequency, PV: percent phenotypic variance explained by a quantitative trait locus (QTL), Known *R* genes/QTL: Known *R* genes and QTL that overlapped with detected QTL. Bold underlined letters mean that nucleotide is associated with lower disease severity or infection type values representing favorable (resistance) alleles. Gene, annotation and function were identified based on the Chinese Spring Reference Genome v1.0 assembly from EnsemblPlants (https://plants.ensembl.org/), UniProt (https://www.uniprot.org/), InterPro (https://www.ebi.ac.uk/interpro/), and searching the literature for functions related to plant stress response. Comparison of QTL physical bp positions to known *R* genes and QTL was conducted using a Chinese Spring v1.0 basic local alignment search tool (https://wheat‐urgi.versailles.inra.fr/Seq‐Repository/BLAST).

^a^ Position in mega base pairs (Mb) is to the right of the underscore in the SNP ID.

### 3.4 Allele pyramiding results

Five major QTLs were evaluated to confirm allelic effect from favorable alleles (Table S9). Tukey's honest significant difference test was utilized to compare stacking effects of resistant alleles for a given trait (Table S10; Figure S4). For Plains 2023, for both the LR and YR datasets, lines progressively increased in rust resistance with each added allele. For P23_LR, lines were 69.7% and 88.3% more resistant with one and two of the favorable alleles, respectively. For P23_YR, lines were 70.8% more resistant with one of the favorable alleles. Plains 2023 LR and YR datasets with the maximum number of favorable alleles from their tested QTL were 93.6% and 84.4% more resistant on average compared to the null group, respectively (Table S10; Figure S4).

### 3.5 Candidate gene identification

A candidate gene search based on gene annotation was conducted using Chinese Spring reference genome v1.0 within the International Wheat Genome Sequencing Consortium genome assembly. Using all QTL and datasets, 16 annotated candidate genes were discovered. Eight genes were discovered from only YR data and seven genes from only LR data (Table S8). One gene, *TraesCS2A02G052500*, was discovered from both LR and YR data. Of these candidate genes, only six were found among major QTL (Table 2). *TraesCS1A02G145300, TraesCS2A02G052500*, and *TraesCS2B02G042700* were the candidate genes discovered for major QTL *QYr.1A.1*, *QLrYr‐2A.1*, and *QLr‐2B.1*, respectively. In addition, *TraesCS2D02G020100*, *TraesCS6A02G381800*, and *TraesCS7B02G127800* were identified on major QTL *QLr‐2D.1*, *QLr‐6A.2*, and *QLr‐7B.1*, respectively (Table 2). Furthermore, 24 known rust *R* genes and QTL overlapped with 10 of our QTLs (including *Yr‐1A.1* that overlaps with *YrHA* and *QLrYr‐2A.1* that overlaps with *YrR61*, *Lr17a*, and *QYrtm.pau‐2A*) (Table S8).

Across major and minor detected QTL, six candidate genes were directly associated with biotic stress resistance or response. For major QTL, *TraesCS2A02G052500* discovered from *QLrYr‐2A.1* was associated with effector‐triggered immunity (Yoo et al., 2020), *TraesCS2B02G042700* discovered from *QLr‐2B.1* was associated with immune response (Kim et al., 2011), *TraesCS2D02G020100* discovered from *QLr‐2D.1* was associated with slower growth of fungal pathogens (Sawano et al., 2007), and *TraesCS7B02G127800* discovered from *QLr‐7B.1* was associated with plant biotic stress response (Sood et al., 2014). As for minor QTL, *TraesCS6D02G392700* discovered from *QLr‐6D.1* was associated with controlling pathogen infection response (Li et al., 2022), and *TraesCS7D02G146000* discovered from *QYr‐7D.1* was associated with response to pathogenic microbes (Chandrasekar et al., 2022; Perrot et al., 2022).

## 4 DISCUSSION

Discovering novel LR and YR resistance is a continuous process as *Pt* and *Pst* evolve to overcome currently effective *R* genes. To fully take advantage of our germplasm, this study was conducted to identify novel genetic resistance loci for LR and YR in soft red winter wheat using GWAS. The panel that we evaluated consisted of advanced lines adapted to the southeastern United States climate, so any QTL detected in this study can likely be used by wheat breeding programs in the southeastern United States to help raise LR and YR resistance for this region.

For correlations between LR IT data collected in the greenhouse across years, these values (*r* = 0.66–0.78) were within the range of correlation for seedlings in a study by Sapkota et al. (2019) (*r* = 0.62–0.83). Comparing pairwise correlations across years for LR in Plains, these values (*r* = 0.31–0.43) are lower than Sapkota et al. (2020) (*r* = 0.80) in Plains and Gerard et al. (2018) for their location with the lowest value (*r* = 0.61), La Plata, Argentina. LR disease pressure in both Plains and Williamson varied more from year to year, possibly due to temperature or change in *Pt* isolate composition. While Plains, Williamson, and greenhouse LR *H* ^2^ values calculated for this study ranged from 0.48 to 0.9, 0 to 0.51, and 0.62 to 0.86, respectively, Sapkota et al. (2020) reported 0.82–0.87, 0.85, and 0.94 for those same locations, respectively, using a recombinant inbred line population. Different *Pt* races could have been prevalent over time that affected these values. *Pt* isolate MFGKG was also used in Sapkota et al. (2019), and they derived 0.93 *H* ^2^ from their diversity panel versus the maximum 0.86 *H* ^2^ calculated at the seedling level for this study. This means that genetic causes are more likely to have a stronger influence on the phenotypes of these different panels when inoculated with MFGKG and that MFGKG should be used to screen other populations for LR response.

LD can help us group SNPs together into QTL as well as help us detect markers for causal variants to disease response (Joiret et al., 2019; Otyama et al., 2019). For this study, Bonferroni correction resulted in a stringent threshold where each significant QTL only had one SNP. This threshold limited how much LD decay could group detected SNPs into more inclusive QTL. Under less stringent thresholds, LD decay could have been used to group more of these SNPs together, allowing us to search for candidate genes under larger physical base pair distance intervals. Using −log10(*p*) > 4, however, SNPs were considered significant on a larger number of chromosomes, so we applied a higher threshold to lessen the likelihood of false positives (data not shown). LD decay can still be applied for breeding purposes, though. A shorter LD decay distance of the D genome (∼98 Kb) implies higher resolution can be reached more easily for discovering marker trait associations and fine mapping, which means discovering marker trait associations should be more difficult on the B genome (567 kb) (Otyama et al., 2019). Even though LD decay was not applied to define more inclusive QTL, these results could be used for marker‐assisted selection for further study.

Twenty‐six significant QTLs for YR and LR resistance were discovered in this study from at least two GWAS models across all datasets, including nine major QTLs with 10.1%–30.8% PV. Of our major QTL, two of them, *QYr‐1A.1* and *QLrYr‐2A.1*, overlapped with known rust *R* genes or QTL. *QLrYr‐2A.1* was detected from P19_LR, P22_YR, and W22_YR, with PV ranging from 15.9 to 30.8%. *QYr‐1A.1* (251.38 Mb), detected in P23_YR with up to 14.99% PV, overlapped between *wmc469* (242.83 Mb) and *gwm497* (551.15 Mb), the flanking markers of *Yr* gene *YrHA*. *YrHA* is a *Yr* gene that has shown resistance to predominant *Pst* races in China (Ma et al., 2013). *QLrYr‐2A.1* (20.86 Mb) overlapped between flanking markers of *YrR61*, *Lr17a*, and *QYrtm.pau‐2A*. This is interesting considering that rust *R* genes can cluster together, like *Lr46‐Yr29* (Spychała et al., 2024) and *Lr37‐Yr17‐Sr38* (Helguera et al., 2003). *YrR61*, a major *Yr* gene derived from Pioneer 26R61, was detected in Plains, GA, and Williamson, GA (Hao et al., 2011); *Lr17a* was an all‐stage resistance gene derived from cultivars Klein Lucero and Maria Escobar (Bremenkamp‐Barrett et al., 2008); and *Qyrtm.pau‐2A* was an adult plant resistance QTL derived from line pau14087 (Chhuneja et al., 2008). *Lr17a* has been considered a defeated gene in the US Midwest, but Bremenkamp‐Barrett et al. (2008) supported using its cosegregating markers to pyramid it with other *Lr* genes. *QYrtm.pau‐2A* explained up to 12% PV for YR DS in pooled data from field trials in India in 2004, 2005, and 2007 (Chhuneja et al., 2008). Genotypic validation of *YrHA*, *YrR61*, and *Lr17a* needs to be performed on this population, as these are the known *R* genes with molecular markers that overlapped with detected major QTL *QYr‐1A.1* and *QLrYr‐2A.1*. *YrHA* should be validated with markers *wmc469* and *gwm497* (Ma et al., 2013); *YrR61* should be validated with markers *barc124* and *gwm359* (Hao et al., 2011); and *Lr17a* should be validated with markers *wmc407* and *gwm614* (Bremenkamp‐Barrett et al., 2008). In addition, eight minor QTLs overlapped with 18 known rust *R* genes or QTL (Table S8) (Agenbag et al., 2012; Chen et al., 2012; Christopher et al., 2013; Hou et al., 2015; Lan et al., 2014; F. Liu et al., 2007; J. Liu et al., 2013; Z. Liu et al., 2014; Mebrate et al., 2008; Melichar et al., 2008; Ren et al., 2012; A. Singh et al., 2012; Skowrońska et al., 2020; Tian et al., 2011; Yan et al., 2023; Yin et al., 2006; Zhou, Han, et al., 2015; Zhou, Zhang, et al., 2015). The five GAPIT models in this study did not all detect the same SNPs, which can reflect differences in how each model handles population structure and kinship (Sandhu et al., 2024). Of the datasets in which significant SNPs were detected, FarmCPU and GLM detected significant SNPs the most frequently. However, GLM can lead to numerous false positive results (Sandhu et al., 2024). FarmCPU, on the other hand, was intended to include both random and fixed effects to control false positives, which may explain why it detected significant SNPs more frequently. FarmCPU may be the ideal model for this study because it is also supposed to exclude the confounding relationship between kinship and molecular markers to be more likely to detect true positives (X. Liu et al., 2016). CMLM and MLM have been considered less efficient at identifying true positives. With these models, SNPs detected in a Fusarium head blight study on this same diversity panel were significant at a lower threshold of −log10(*p*) > 4, but they were insignificant when applying Bonferroni correction (Ghimire et al., 2022). Thus, FarmCPU can be considered the most effective for these data.

Significant negative correlations were observed for two datasets between the phenotype data and the number of resistant alleles for each line (P23_LR: *r* = ‐0.48, *p* < 0.001; P23_YR: *r* = −0.35, *p* < 0.001). These correlations showed that pyramiding resistant alleles can help increase resistance to rust pathogens. From our study, combining two favorable alleles from two (P23_YR) or three (P23_LR) major QTL reduced DS by up to 84.4% and 93.6%, respectively. This finding has been reported in many previous studies. F. Wang et al. (2023) showed pyramiding *Yr18*, *Yr28*, and *Yr36* together resulted in significantly lower ITs than other combinations. This was especially true in comparison to susceptible control variety SY95‐71 for both seedling (3.3 vs. 8.2) and adult plant stages (1.6 vs. 8.5) (F. Wang et al., 2023). Ward et al. (2021) observed similar results for YR, demonstrating lines from their southeastern elite panel with all resistant alleles from three QTLs on chromosomes 2A, 3B, and 4B had the lowest average DS compared to other allele combinations. Similarly for LR DS, lines with pyramided *Lr2a*, *Lr16*, *Lr34*, and *Lr46* were consistently in the lowest significance group across environments (Bokore et al., 2022). Recently, Ghimire et al. (2022) evaluated the SWAMP panel for efficacy of allele pyramiding on Fusarium head blight resistance and revealed that more resistant alleles consistently lead to higher resistance. However, the highest increase in resistance they observed was 55% from lines with only null alleles, while our highest increase was 93.6%. This could be due to Fusarium head blight resistance being inherited quantitatively, while LR and YR can exhibit either qualitative or quantitative resistance (Ghimire et al., 2022; Mapuranga et al., 2022).

Candidate genes, mostly related to stress resistance, were found for six out of the nine major QTLs. *TraesCS1A02G145300* (*QYr‐1A.1*), annotating for a glycine‐aspartate‐serine‐leucine‐type esterase/lipase protein, was associated with lipid metabolism or hydrolase activity (Dash et al., 2016; X. Yang et al., 2022). Vishwakarma et al. (2023) showed that lipid metabolism helped biosynthesize secondary metabolites, which helped with stem rust resistance in wheat line HW2004, carrying resistance gene *Sr24*. Activity for different types of hydrolases was listed as functions for differentially expressed genes for LR and stem rust (peptidyl‐tRNA hydrolase) as well as YR (glycoside hydrolase, metal‐dependent hydrolase, amidohydrolase 3, and peptidyl‐tRNA hydrolase) (Pal et al., 2022). *TraesCS2A02G052500* (*QLrYr‐2A.1*), annotated for a lysine histidine transporter, was associated with sulfur deficiency response in *Medicago truncatula* (Wipf et al., 2014), and this gene is also annotated for an amino acid permease 3 associated with effector‐triggered immunity and nematode parasitism in *Arabidopsis thaliana* (Marella et al., 2013; Yoo et al., 2020). Proper sulfur input can reduce likelihood of stem rust infection (Singh, 2015), and sulfur fertilizer can be combined with *Thiobacillus* bacteria to lower severity of Take‐all DS in wheat (Ghadamkheir et al., 2020). *TraesCS2B02G042700* (*QLr‐2B.1*) annotated for a disease resistance protein group and NB‐ARC domain‐containing CC‐NBS‐LRR *R* proteins were involved with potato late blight resistance protein R1 and immune response, respectively (Ballvora et al., 2002; Kim et al., 2011). *Ne2* is a gene that helps produce wheat hybrid necrosis, encodes a CC‐NBS‐LRR *R* protein, and its allele, *Ne2^m^*, and *Lr13* are the same gene (Si et al., 2021). Fusion protein Ta7ANB‐ARC–NPR1 was involved with negative regulation of stem rust resistance (X. Wang et al., 2020). *TraesCS2D02G020100* (*QLr‐2D.1*) annotated for an apoplastic protein and an antifungal protein were associated with salt stress response in rice and growth reduction of fungal pathogens, respectively (Sawano et al., 2007; L. Zhang et al., 2009). When *Lr39/41* was involved in an incompatible interaction against early LR infection, gene *TaLTP3* in the plant apoplastic space was upregulated. Overexpression of *TaLTP3* resulted in higher LR resistance (Zhao et al., 2021). *TraesCS6A02G381800* (*QLr‐6A.2*) was annotated for a W2 domain‐containing protein that was associated with lower expression under heat, cold, salt, and drought stresses (Habibpourmehraban et al., 2022). High temperature adult plant YR resistance is durable, not *Pst* race specific, and is recommended for use worldwide, considering *Pst* races are adapting to warmer climates (Miedaner & Juroszek, 2021). Miedaner and Juroszek (2021) also argue further study should be conducted on how drought stress affects wheat resistance gene expression because there is not as much research on that compared to temperature effects on *Yr* genes. *TraesCS7B02G127800* (*QLr‐7B.1*), annotated for a Myb‐like DNA binding domain family, was associated with disease resistance in plants (Sood et al., 2014). An MYB‐DNA binding domain was conserved in a late elongated hypocotyl gene in wheat, *TaLHY*, which is involved in resistance against YR infection (Z. Zhang et al., 2015).

In this study, we were able to evaluate the SWAMP panel under greenhouse conditions across years and field conditions across years and locations. While two of our major QTLs and eight of our minor QTLs overlapped with known rust *R* genes and QTL, we still discovered 16 novel QTLs from our GWAS results. These novel QTLs could be an asset in SE wheat breeding programs to combat emerging *Pt* and *Pst* races and safeguard SE US wheat production.

## AUTHOR CONTRIBUTIONS

**John W. Bagwell**: Data curation; formal analysis; investigation; methodology; software; validation; visualization; writing—original draft; writing—review and editing. **Mohamed Mergoum**: Conceptualization; funding acquisition; methodology; project administration; resources; supervision; writing—review and editing. **Madhav Subedi**: Data curation; investigation; writing—review and editing. **Suraj Sapkota**: Data curation; investigation; writing—review and editing. **Bikash Ghimire**: Methodology; software; supervision; writing—review and editing. **Benjamin Lopez**: Investigation; methodology; writing—review and editing. **James W. Buck**: Conceptualization; funding acquisition; methodology; project administration; resources; supervision; writing—review and editing. **Bochra A. Bahri**: Conceptualization; funding acquisition; methodology; project administration; resources; supervision; writing—review and editing.

## CONFLICT OF INTEREST STATEMENT

The authors declare no conflicts of interest.

## Supporting information



Supplemental Table S1. Genotype data for 230 soft wheat association mapping panel lines.

Supplemental Table S2. Phenotypic values used in phenotypic data analysis, allelic effect comparison for statistically significant QTL, and to create scatter plots for allele pyramiding evaluation for major QTL.

Supplemental Table S3. Best linear unbiased estimates used for genome‐wide association study on the soft wheat association mapping panel.

Supplemental Table S4. Linkage disequilibrium values for chromosomes, genomes, and the whole genome of the soft wheat association mapping panel.

Supplemental Table S5. Results from Fisher's least significant difference (LSD) test for leaf rust and stripe rust disease severity and infection types across environments for the soft wheat association mapping panel.

Supplemental Table S6. Correlations between trait replicates and averages for leaf rust and stripe rust response across growth stages and environments.

Supplemental Table S7. Analysis of variance (ANOVA) results for leaf rust and stripe rust results across environments from the soft wheat association mapping panel.

Supplemental Table S8. Raw QTL table made from SNPs detected from at least two GWAS models with ‐log10(p) values over 6.44 for leaf rust and stripe rust response.

Supplemental Table S9. Estimation of allelic effect for eight major quantitative trait loci (QTL).

Supplemental Table S10. Stacking effects of resistant alleles contributing to leaf rust and stripe rust response in the soft wheat association mapping panel.

Supplemental Figure S1. Frequency distributions of the soft wheat association mapping panel for leaf rust disease severity across environments and years.
Supplemental Figure S2. Frequency distributions of the soft wheat association mapping panel for stripe rust disease severity across environments and years.
Supplemental Figure S3. Frequency distributions of infection types for leaf rust for 2022 dates (a) 1 and (b) 2 and (c) 2023 and (d) stripe rust in 2024.
Supplemental Figure S4. Scatter plots showing phenotypic averages vs. the number of resistant alleles for (a) Plains 2023 leaf rust severity, and (b) Plains 2023 stripe rust severity.

## Data Availability

Genotypic data can be found in Table S1, and phenotypic data can be found in Table S2.
