# Supplementary material for: Discovering leaf and stripe rust resistance in soft red winter wheat through genome‐wide association studies
Source: Plant Genome. 2025 Jun 11;18(2):e70055. doi: 10.1002/tpg2.70055 (PMC12152529; doi:10.1002/tpg2.70055)
Supplement: Supplementary file 11 — Supplemental Figure S1. Frequency distributions of the soft wheat association mapping panel for leaf rust disease severity across environments and years. Supplemental Figure S2. Frequency distributions of the soft wheat association mapping panel for stripe rust disease severity across environments and years. Supplemental Figure S3. Frequency distributions of infection types for leaf rust for 2022 dates (a) 1 and (b) 2 and (c) 2023 and (d) stripe rust in 2024. Supplemental Figure S4. Scatter plots showing phenotypic averages vs. the number of resistant alleles for (a) Plains 2023 leaf rust severity, and (b) Plains 2023 stripe rust severity. [file TPG2-18-e70055-s007.docx]

Manuscript title: Discovering leaf and stripe rust resistance in soft red winter wheat through genome-wide association studies

Authors: John W. Bagwell^1#^, Mohamed Mergoum^2^, Madhav Subedi^3^, Suraj Sapkota^4^, Bikash Ghimire^5^, Benjamin Lopez^2^, James W. Buck^5^, Bochra A. Bahri^5*^

Affiliations: (1) Department of Forestry and Environmental Resources, North Carolina State University, Raleigh, NC 27606, (2) Department of Crop and Soil Sciences, University of Georgia, Griffin Campus, Griffin, GA 30223, (3) Cornell Institute of Biotechnology, Cornell University, Ithaca, NY 14853, (4) USDA-ARS, Small Grains and Potato Germplasm Research Unit, Aberdeen, ID 83210, (5) Department of Plant Pathology, University of Georgia, Griffin Campus, Griffin, GA 30223.

^#^ Current address: Department of Forestry and Environmental Resources, North Carolina State University, Raleigh, NC 27606

^*^ Corresponding author

Number of pages in this document: 6

Number of supplemental figures: 4

Number of supplemental tables: 10 (See corresponding Excel file for supplemental tables).


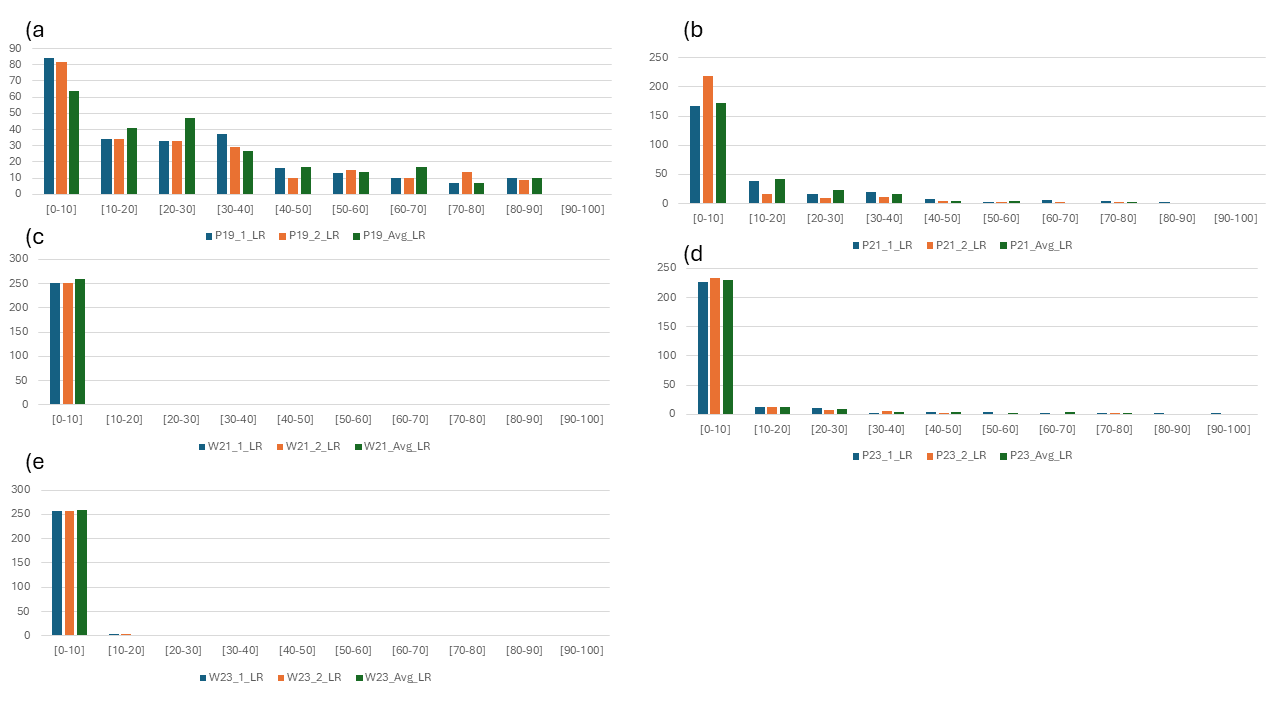


Supplemental Figure S1. Frequency distributions of the soft wheat association mapping panel for leaf rust disease severity across environments and years. (a) Plains 2019, (b) Plains 2021, (c) Williamson 2021, (d) Plains 2023, and (e) Williamson 2023.


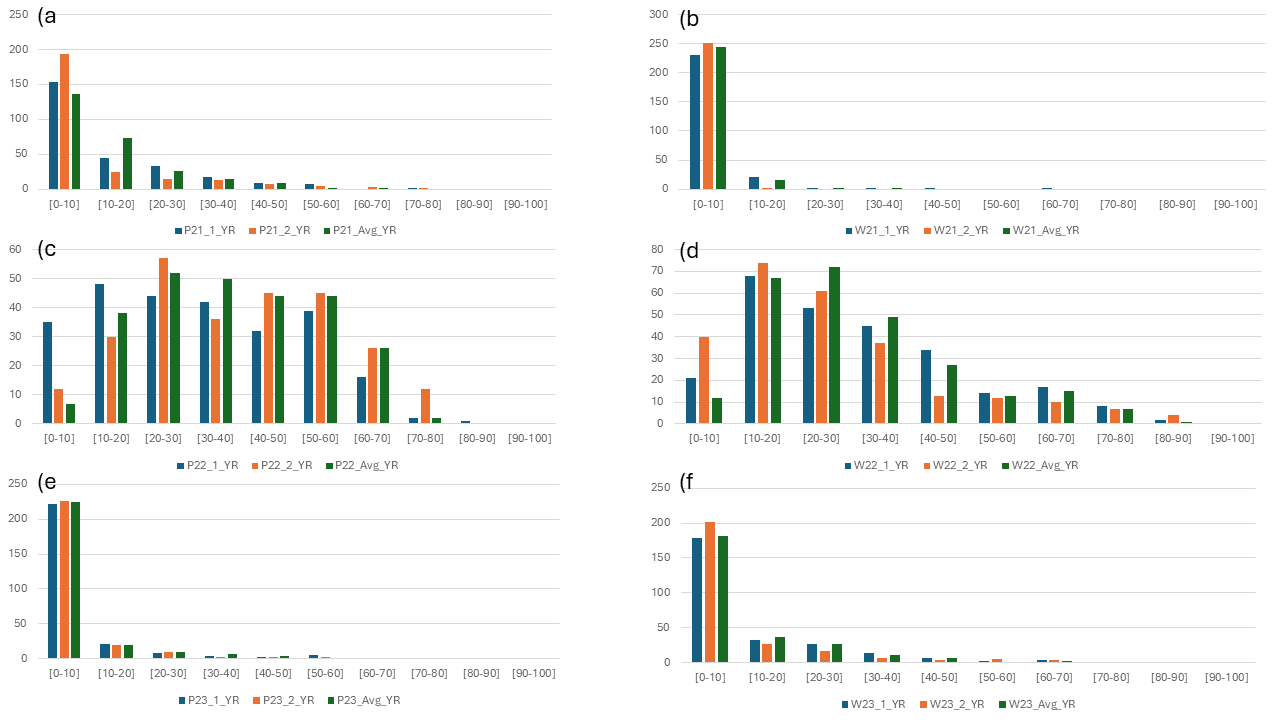


Supplemental Figure S2. Frequency distributions of the soft wheat association mapping panel for stripe rust disease severity across environments and years. (a) Plains 2021, (b) Williamson 2021, (c) Plains 2022, (d) Williamson 2022, (e) Plains 2023, and (f) Williamson 2023.


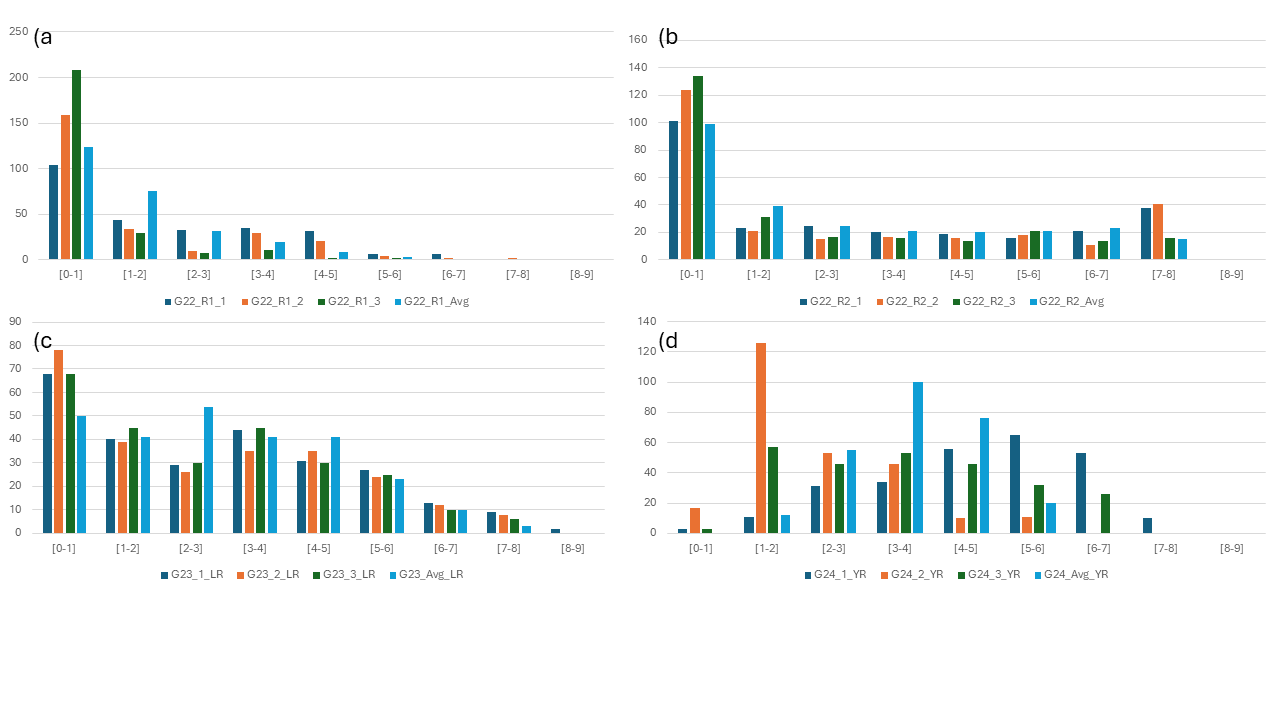


Supplemental Figure S3. Frequency distributions of infection types for leaf rust for 2022 dates (a) 1 and (b) 2 and (c) 2023 and (d) stripe rust in 2024.


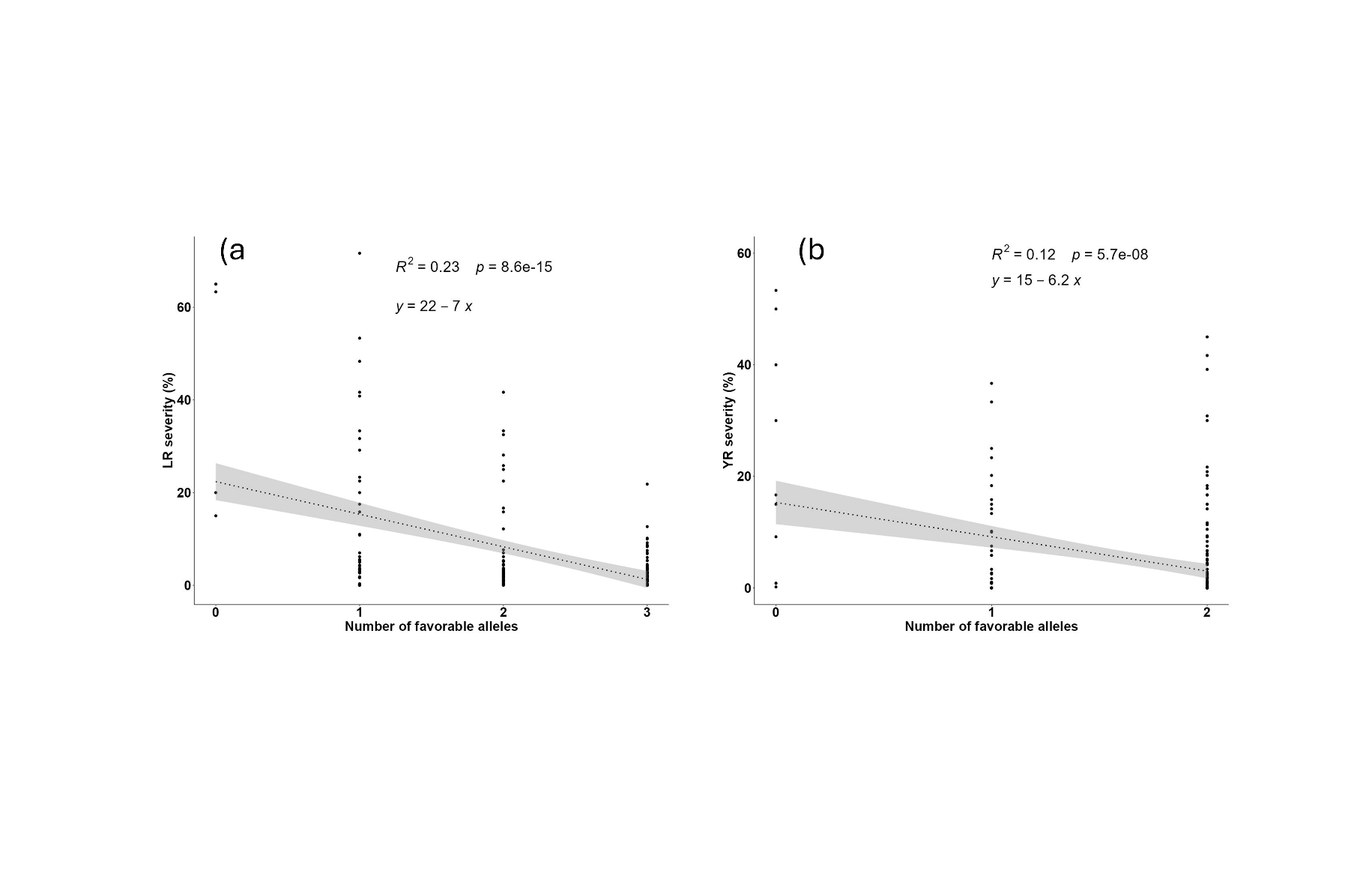


Supplemental Figure S4. Scatter plots showing phenotypic averages vs. the number of resistant alleles for (a) Plains 2023 leaf rust severity, and (b) Plains 2023 stripe rust severity. All regressions were statistically significant (*P* < 0.001).

Supplemental Table S1. Genotype data for 230 soft wheat association mapping panel lines

Supplemental Table S2. Phenotypic values used in phenotypic data analysis, allelic effect comparison for statistically significant QTL, and to create scatter plots for allele pyramiding evaluation for major QTL.

Supplemental Table S3. Best linear unbiased estimates used for genome-wide association study on the soft wheat association mapping panel.

Supplemental Table S4. Linkage disequilibrium values for chromosomes, genomes, and the whole genome of the soft wheat association mapping panel.

Supplemental Table S5. Results from Fisher's least significant difference (LSD) test for leaf rust and stripe rust disease severity and infection types across environments for the soft wheat association mapping panel.

Supplemental Table S6. Correlations between trait replicates and averages for leaf rust and stripe rust response across growth stages and environments.

Supplemental Table S7. Analysis of variance (ANOVA) results for leaf rust and stripe rust results across environments from the soft wheat association mapping panel.

Supplemental Table S8. Raw QTL table made from SNPs detected from at least two GWAS models with -log10(p) values over 6.44 for leaf rust and stripe rust response.

Supplemental Table S9. Estimation of allelic effect for eight major quantitative trait loci (QTL).

Supplemental Table S10. Stacking effects of resistant alleles contributing to leaf rust and stripe rust response in the soft wheat association mapping panel.
